# Prediction of postoperative outcomes using intraoperative hemodynamic monitoring data

**DOI:** 10.1038/s41598-017-16233-4

**Published:** 2017-11-27

**Authors:** Varesh Prasad, Maria Guerrisi, Mario Dauri, Filadelfo Coniglione, Giuseppe Tisone, Elisa De Carolis, Annagrazia Cillis, Antonio Canichella, Nicola Toschi, Thomas Heldt

**Affiliations:** 1 Harvard-MIT Health Sciences and Technology Program, Massachusetts Institute of Technology, Cambridge, MA USA; 2 Institute for Medical Engineering and Science, Massachusetts Institute of Technology, Cambridge, MA USA; 3 Medical Physics Section, Department of Biomedicine and Prevention, University of Rome “Tor Vergata”, Rome, Italy; 4 Department of Clinical Science and Translational Medicine, University of Rome “Tor Vergata”, Rome, Italy; 5 Department of Emergency and Critical Care Medicine, Pain Medicine and Anaesthesiology, University Hospital “Tor Vergata”, Rome, Italy; 6 University “Our Lady of Good Counsel”, Tirana, Albania; 7 Department of Experimental Medicine and Surgery, University of Rome “Tor Vergata”, Rome, Italy; 8 Department of Radiology, Athinoula A. Martinos Center for Biomedical Imaging, Massachusetts General Hospital and Harvard Medical School, Boston, MA USA; 9 Department of Electrical Engineering and Computer Science, Massachusetts Institute of Technology, Cambridge, MA USA

## Abstract

Major surgeries can result in high rates of adverse postoperative events. Reliable prediction of which patient might be at risk for such events may help guide peri- and postoperative care. We show how archiving and mining of intraoperative hemodynamic data in orthotopic liver transplantation (OLT) can aid in the prediction of postoperative 180-day mortality and acute renal failure (ARF), improving upon predictions that rely on preoperative information only. From 101 patient records, we extracted 15 preoperative features from clinical records and 41 features from intraoperative hemodynamic signals. We used logistic regression with leave-one-out cross-validation to predict outcomes, and incorporated methods to limit potential model instabilities from feature multicollinearity. Using only preoperative features, mortality prediction achieved an area under the receiver operating characteristic curve (AUC) of 0.53 (95% CI: 0.44–0.78). By using intraoperative features, performance improved significantly to 0.82 (95% CI: 0.56–0.91, P = 0.001). Similarly, including intraoperative features (AUC = 0.82; 95% CI: 0.66–0.94) in ARF prediction improved performance over preoperative features (AUC = 0.72; 95% CI: 0.50–0.85), though not significantly (P = 0.32). We conclude that inclusion of intraoperative hemodynamic features significantly improves prediction of postoperative events in OLT. Features strongly associated with occurrence of both outcomes included greater intraoperative central venous pressure and greater transfusion volumes.

## Introduction

In many acute and chronic diseases, major surgery like organ transplantation is the only option for curative treatment. Such surgeries may commonly result in high postoperative incidences of major adverse events. Preoperative evaluation is widely used in many surgical contexts to identify patients at greatest risk in the peri- and postoperative period and to design perioperative strategies to mitigate these risks^1^. However, preoperative risk assessment is not always possible or even accurate. In orthotopic liver transplantation (OLT), for example, predicting postoperative outcomes preoperatively remains difficult. Efforts to use simple measures of a recipient’s level of preoperative disease severity, *e*.*g*., model for end-stage liver disease (MELD) and Child-Pugh scores, have shown limited usefulness in predicting postoperative mortality and other outcomes^2–6^.

Conversely, intraoperative measures, in particular hemodynamic measures, have been shown to be related to various postoperative outcomes in OLT and other major abdominal surgeries. For instance, increased variability of intraoperative mean arterial blood pressure (ABP) is associated with 1-month mortality after OLT^7^, and lower systolic and mean ABP^8,9^ and cardiac index^9^ are associated with postoperative acute renal failure (ARF). In contrast, lower central venous pressure (CVP) is associated with reduced blood loss and transfusion^10,11^, as well as fewer pulmonary complications^12^, though it is also associated with renal impairment and 30-day mortality^13^. In major abdominal surgeries in general, lower CVP^14,15^ and stroke volume variation (SVV)^16^ are both associated with reduced mortality and morbidity.

These results suggest that mining intraoperative variables might aid risk stratification and guide prompt and appropriate peri- and postoperative care. Notably, hemodynamics are also modifiable in nature; a strong association between hemodynamic variables and postoperative outcomes would therefore present opportunities to further study potential causative relationships involving intraoperative management. Despite the importance of intraoperative management in OLT in particular and most high-risk surgeries, institutional practices vary widely, and accepted standards for intraoperative care are lacking^2,17–20^.

Continuous high-resolution physiological waveforms are commonly recorded and displayed during major surgeries; however, they have rarely been archived. Yet, archiving such datasets would enable retrospective analysis, including mining for important associations that may inform future prospective studies and ultimately, clinical practice. With the increasing availability of archiving solutions that collect and store multimodal high-resolution waveform data, a much wider range of hemodynamic variables can now be mined compared to prior studies. Such work, though, may suffer from technical challenges. A comprehensive hemodynamic dataset is likely to suffer from multicollinearity, which typically causes unreliable estimates in predictive models^21^. Moreover, in the context of OLT, the number of annual procedures is typically low even at major tertiary care centers. The low number of patients from whom data can be sourced in turn limits the number of variables that can be explored through multivariate methods with accuracy and reliability^22^.

This work seeks to address these challenges. It describes the extraction and archiving of intraoperative physiological waveform data from OLT patients at a single tertiary care center and a machine learning approach optimized for evaluating the performance of a large and diverse set of hemodynamic variables in predicting postoperative 180-day mortality and ARF after OLT. Our overall hypothesis is that the use of intraoperative features can significantly improve these predictions. We investigate this hypothesis using a data mining approach to also help identify the main features driving the improvement.

## Results

### Cohort

**Figure 1:**
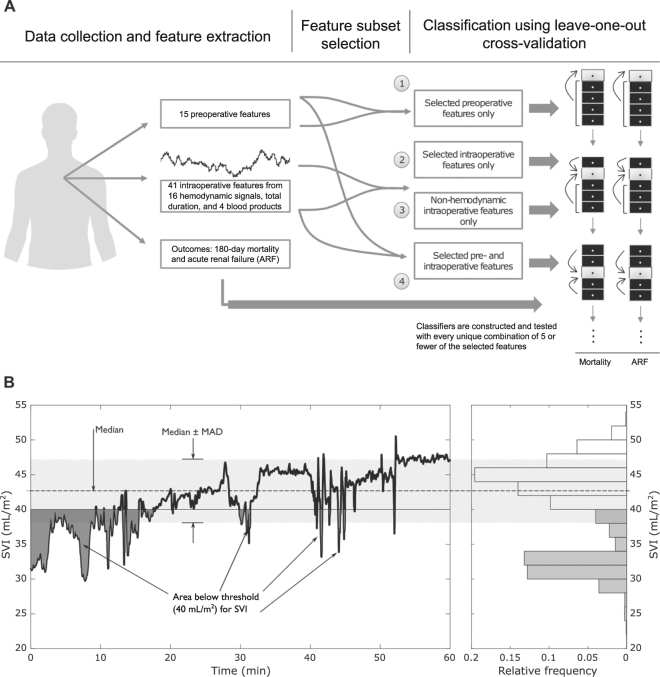
(**A**) Overview of data collection and outcome prediction procedure. Data were collected and extracted from medical records and intraoperative hemodynamic monitors. Prediction of each outcome was carried out in four separate tasks with different groups of features after performing subset selection within each feature group. In each task, logistic regression classifiers were constructed with every combination of 5 or fewer features and leave-one-out cross-validation was used for training and testing. (**B**) Illustration of the three features extracted from each continuously computed hemodynamic signal. In addition to the median and median absolute deviation (MAD), the integrated area of the signal relative to a normal threshold (either above or below the threshold) was computed. For the stroke volume index (SVI) signal here, the area below 40 mL/m^2^ was computed. Left: a 60-minute portion of one patient’s SVI waveform. Right: the histogram of this signal’s values.

The first step in our procedure (Fig. 1a) involved collection of relevant pre-, intra-, and postoperative data. In total, we reviewed records from 101 patients, including 14,291 overall hours of intraoperative waveform data. Table 1 summarizes demographic and preoperative clinical information from this cohort and compares them across outcomes. Preoperative serum albumin was significantly lower in both 180-day mortality (P = 0.007, Wilcoxon rank-sum test) and ARF groups (P = 0.009) *versus* respective controls. MELD score was significantly greater in the ARF group *versus* controls (P = 0.006). Sixty-two records had sufficient data to be included in prediction tasks for mortality (see Methods for inclusion criteria). Among these records, eleven (17.7%) cases of 180-day mortality were present, with a median survival time of 11 days. Due to variability in the availability of outcome information, a largely overlapping but partially distinct set of 62 records had sufficient data for ARF prediction tasks. Thirteen ARF cases (21.0%) were present in this group.

**Table 1:** Characterization of overall patient population with univariate group differences. Continuous data are presented as median (interquartile range) and tested for differences between groups by the Wilcoxon rank sum test. Categorical data are presented as counts (%) and tested for differences between groups by the chi-squared test.

Characteristic	Survival	N	180-day mortality	N	P-value	No ARF	N	ARF	N	P-value
Age	56 (50–61)	72	57 (51–62)	26	0.729	56 (51–62)	72	56 (46–62)	22	0.421
Male	53 (74.7)	71	18 (72.0)	25	0.795	54 (76.1)	71	15 (71.4)	21	0.667
Weight (kg)	73 (65.0–83.5)	72	75.5 (65.0–84.0)	26	0.961	73 (66.0–82.5)	72	74 (60.0–84.0)	22	0.607
Body Mass Index (kg/m^2^)	25.9 (22.4–28.2)	71	26 (23.0–27.3)	26	0.845	26 (22.7–28.1)	71	24.1 (22.5–27.3)	22	0.292
Diabetes	16 (22.2)	72	8 (30.8)	26	0.385	17 (23.6)	72	7 (31.8)	22	0.44
Hypertension	22 (30.6)	72	7 (26.9)	26	0.728	21 (29.2)	72	6 (27.3)	22	0.864
Smoking	20 (27.8)	72	6 (23.1)	26	0.642	21 (29.2)	72	5 (22.3)	22	0.555
ASA Class										
1	0 (0)	51	0 (0)	18	0.770	0 (0)	51	0 (0)	15	0.690
2	5 (9.8)		1 (5.6)			5 (9.8)		1 (6.7)		
3	40 (78.4)		14 (77.8)			40 (78.4)		11 (73.3)		
4	6 (11.8)		3 (16.7)			6 (11.8)		3 (20.0)		
Child-Pugh Score										
A	17 (30.9)		7 (31.8)			16 (28.1)		5 (29.4)		
B	22 (40.0)		8 (36.4)			24 (42.1)		5 (29.4)		
C	16 (29.1)	55	7 (31.8)	22	0.953	17 (29.8)	57	7 (41.2)	17	0.587
MELD	18 (11–20)	72	17 (11–20)	26	0.977	16 (10–19)	72	20 (16–25)	22	0.006
Piggyback Technique	30 (41.7)	72	7 (28.0)	25	0.226	29 (40.9)	71	7 (31.8)	22	0.448
Marginal Donor	34 (47.2)	72	10 (38.5)	26	0.441	36 (50.0)	72	8 (36.4)	22	0.262
INR	1.49 (1.20–1.80)	72	1.56 (1.30–1.70)	26	0.454	1.48 (1.20–1.73)	72	1.61 (1.39–1.83)	22	0.15
Direct bilirubin (mg/dL)	1.17 (0.48–1.97)	68	0.91 (0.60–2.35)	25	0.845	1.13 (0.48–1.79)	70	1.88 (0.71–4.80)	20	0.066
Total bilirubin (mg/dL)	1.5 (0.59–3.08)	70	1.54 (0.80–2.64)	26	0.378	1.5 (0.66–3.08)	70	1.99 (1.06–4.10)	22	0.144
Albumin (g/dL)	3.20 (2.90–3.60)	72	2.90 (2.35–3.23)	25	0.007	3.20 (2.90–3.60)	72	2.80 (2.40–3.20)	21	0.009
Creatinine (mg/dL)	0.90 (0.80–1.12)	72	0.90 (0.70–1.50)	26	0.984	0.90 (0.80–1.10)	72	1.10 (0.70–2.10)	22	0.131

*ARF: Acute renal failure*.

*MELD: model for end-stage liver disease score*.

*INR: international normalized ratio of prothrombin time*.

*ASA: American Society of Anesthesiologists*.

### Feature extraction and subset selection

Fifteen preoperative and 41 intraoperative features were extracted from available data (Table 2), including three separate features from each hemodynamic waveform (Fig. 1b) designed to capture the central tendency of the waveform (median), the variability of the waveform (median absolute deviation [MAD]), and the overall integrated exposure of the patient to potentially harmful conditions. See Methods and online Supplementary Material 1 for full data collection procedures and feature descriptions.

**Table 2:** Lists of pre- and intraoperative features and inclusion by subset selection into the pre-operative, intra-operative, and combined (pre- and intra-operative) feature sets.

	**Variable (Abbreviation) [unit]**		**Subset inclusion**		
			Only pre-operative features	Only intra-operative features	Combined features
Preoperative features	Age [years]		X		X
	Marginal vs. nonmarginal donor		X		X
	Prothrombin time international normalized ratio (INR)		X		
	Serum direct bilirubin [mg/dL]		X		X
	Serum albumin [g/dL]				
	Serum creatinine [mg/dL]		X		X
	Model for end-stage liver disease score (MELD)				
	Classical vs. piggyback surgical technique		X		X
	Male vs. female sex		X		X
	History of diabetes		X		X
	History of hypertension		X		X
	Present smoking status		X		X
	American Society of Anesthesiologists (ASA) class		X		X
	Body mass index (BMI) [kg/m^2^]				
Non-hemodynamic intraoperative features	Volume of whole blood administered [mL/kg]			X	
	Volume of fresh frozen plasma administered [mL/kg]			X	X
	Volume of platelets administered [mL/kg]			X	X
	Volume of packed red blood cells autotransfused [mL/kg]			X	X
	Overall surgery duration [min]				
Intraoperative hemodynamic variables and extracted features	Systolic arterial blood pressure (SBP) [mmHg]	Median			
		MAD			
		Area below 100 mmHg		X	X
	Central venous pressure (CVP) [mmHg]	Median			
		MAD		X	X
		Area above 5 mmHg		X	X
	Heart rate (HR) [bpm]	Median			
		MAD		X	X
		Area above 100 bpm		X	X
	Peripheral oxygen saturation (SpO_2_) [%]	Median			
		MAD		X	X
		Area below 90%		X	X
	Cardiac function index (CFI) [min^−1^]	Median			
	Max left ventricular contractility (dPmx) [mmHg/s]	Median			
		MAD		X	X
		Area below 642 mmHg/s		X	X
	Extravascular lung water index (ELWI) [mL/kg]	Median			
	Global end-diastolic volume index (GEDI) [mL/m^2^]	Median			
	Global ejection fraction (GEF) [%]	Median			
	Intrathoracic blood volume index (ITBI) [mL/min/m^2^]	Median			
	Pulse-contour cardiac index (PCCI) [L/min/m^2^]	Median			
		MAD		X	X
		Area below 3 L/min/m^2^		X	X
	Pulse pressure variation (PPV) [%]	Median			
		MAD			
		Area above 10%		X	X
	Pulmonary vascular permeability index (PVPI)	Median			
	Stroke volume index (SVI) [mL/m^2^]	Median			
		MAD		X	X
		Area above 40 mL/m^2^		X	X
	Systemic vascular resistance index (SVRI) [dyn·s·cm^−5^·m^2^]	Median			
		MAD		X	
		Area below 1700 dyn·s·cm^−5^·m^2^		X	
	Stroke volume variation (SVV) [%]	Median			
		MAD		X	
		Area above 10%		X	X

Because of the expected collinearities among all the features included, we used a subset selection procedure to identify an initial set of appropriate features with reduced collinearities among them. This step was performed without using the true class labels. For each outcome, we performed four tasks, classifying patients into the outcome (mortality or ARF) group or the control group using (1) only preoperative features, (2) only intraoperative features, (3) pre- and intraoperative features, and (4) only blood product volumes and surgery duration (*i*.*e*., non-hemodynamic intraoperative information).

Subset selection was performed separately for the feature set used for each task. The selected subsets of features for Tasks 1 through 3 are noted in Table 2; all non-hemodynamic features passed the subset selection step for Task 4. For tasks involving only preoperative features, only intraoperative features, combined pre- and intraoperative features, and only non-hemodynamic intraoperative features, we used subsets consisting of 11, 22, 27, and 5 features, respectively (see online Supplementary Material 2, Fig. S2 for details).

### Prediction results

We used logistic regression with leave-one-out cross-validation to predict the binary outcomes in each task. To limit the feature-to-case ratio, we included only up to five features in each classifier and tested every possible combination thereof (*i*.*e*., an exhaustive search approach). Each task then had respective totals of 1,023 (Task 1), 35,442 (Task 2), 101,583 (Task 3), and 32 (Task 4) unique combinations of five or fewer features to be tested. To evaluate classifier performance, we computed the area under the receiver operating characteristic curve (AUC). Because an AUC of 0.7 is approximately the ceiling for performance in previous studies of postoperative mortality prediction in OLT^4,6^, we used 0.7 as a threshold to identify high-performing classifiers. We also computed multivariate odds ratios (OR) from multivariate logistic regression coefficients to describe the associations of features with the odds of each outcome.

### Postoperative 180-day mortality

**Figure 2:**
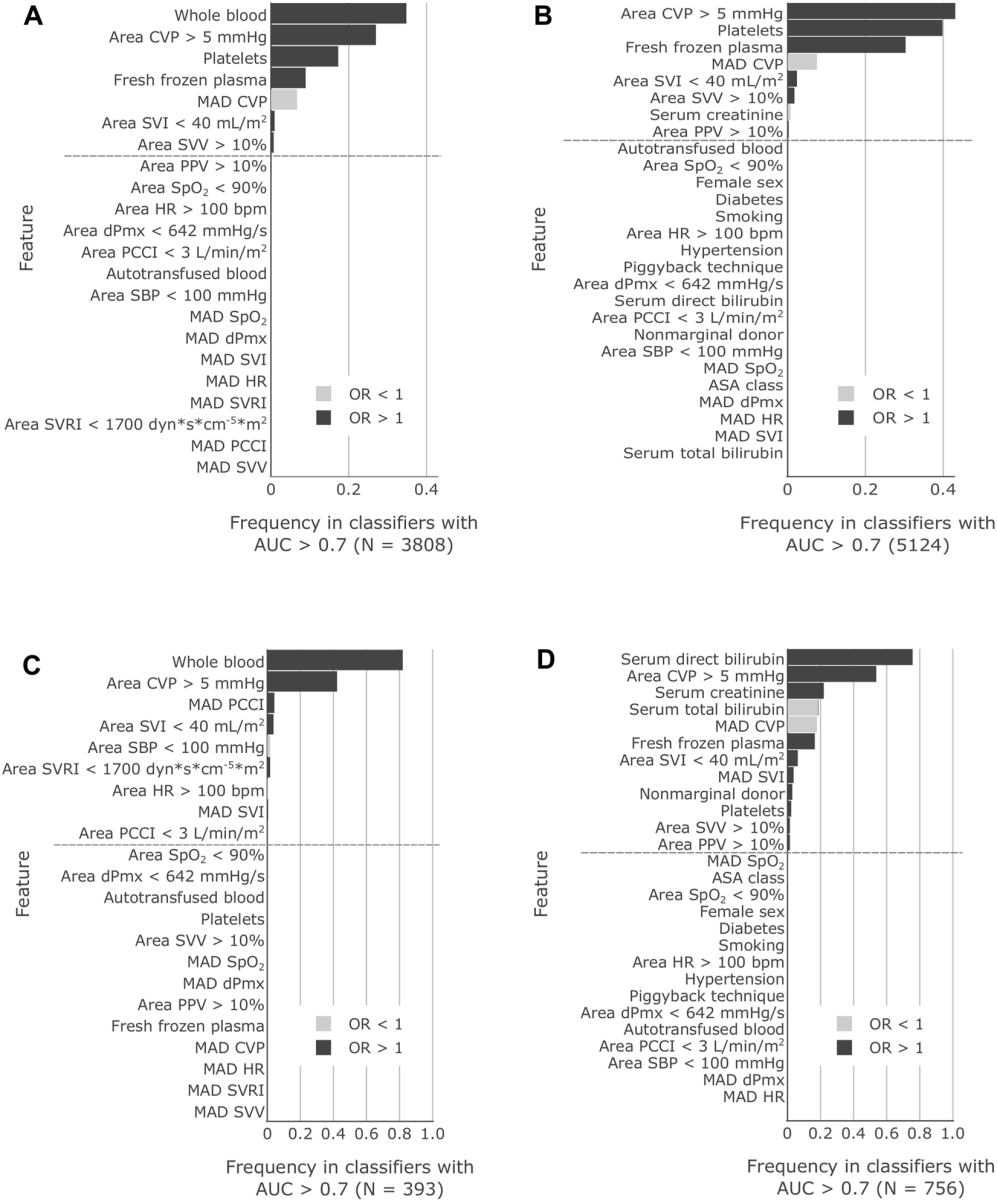
Frequency that features are included at a significant level (P < 0.05) in classifiers with AUC greater than 0.7. (**A**) Results from 180-day mortality classifiers that used only intraoperative features and (**B**) that used both pre- and intraoperative features. (**C**) Results from ARF classifiers that used only intraoperative features and (**D**) that used both pre- and intraoperative features. Dark shading indicates the fraction of classifiers in which the feature was included with odds ratio (OR) greater than 1 and light shading indicates OR less than 1. The monotone nature of each bar’s shading indicates that features always had ORs on the same side of 1, *i*.*e*., they were always associated with risk in the same direction. Features below the dashed line were never included at a significant level.

The best performance was achieved by intraoperative-features-only (maximum AUC = 0.82, 95% CI: 0.56–0.91) and combined preoperative- and intraoperative-features classifiers (maximum AUC = 0.81, 95% CI: 0.64–0.94, P = 0.93 compared to maximum intraoperative-only AUC) (Table 3). Both of these classifiers significantly outperformed the best preoperative-features-only classifier (maximum AUC = 0.53, 95% CI: 0.44–0.78, P = 0.001 and 0.003, respectively). Figures 2a,b display the features included in the intraoperative-features-only and combined-features classifiers with AUC greater than 0.7. Note that many of these features do not appear in the single best classifier described in more detail in Table 3 (or may appear in that classifier without statistical significance), but their presence in other classifiers with similarly high AUCs indicates that they retain significant predictive capability. For each feature, the fractional bar shading indicates the fraction of classifiers in which the feature’s OR was greater than (black) and less than (gray) 1.0. The monotone nature of each bar’s color indicates that ORs always show association in only one direction in all classifiers. For example, with the exception of MAD CVP and serum creatinine, most features showed positive correlation with occurrence of mortality. Out of all preoperative features, serum creatinine was the only variable included.

**Table 3:** AUCs and odds ratios for the best mortality and ARF classifiers.

Outcome	Possible features	Best AUC (95% CI)	Features included in best classifier	Odds ratios (95% CI)	P-value
180-day Mortality	Preoperative	0.53 (0.44–0.78)	Smoking	0.204 (0.024–1.739)	0.146
			Hypertension	0.572 (0.107–3.068)	0.514
			Nonmarginal donor	0.724 (0.189–2.772)	0.637
	Intraoperative	0.82 (0.56–0.91)	MAD dPmx	0.987 (0.968–1.007) s/mmHg	0.201
			MAD CVP	0.399 (0.163–0.979) mmHg^−1^	0.045
			RBC	1.095 (1.023–1.171) kg/mL	0.008
			Area CVP > 5 mmHg	1.001 (1.000–1.001) (mmHg·min)^−1^	0.048
	Combined	0.81 (0.64–0.94)	Platelets	1.276 (1.036–1.571) kg/mL	0.022
			Serum creatinine	0.026 (0.000–1.646) dL/mg	0.085
			Area SVI < 40 mL/m^2^	1.002 (1.000–1.003) (mL·m^−2^·min)^−1^	0.024
			MAD dPmx	0.989 (0.968–1.010) s/mmHg	0.288
			Area CVP > 5 mmHg	1.001 (1.000–1.001) (mmHg·min)^−1^	0.009
	Blood product volumes and duration	0.75 (0.56–0.93)	Whole blood	1.086 (1.030–1.145) kg/mL	0.002
Acute Renal Failure	Preoperative	0.72 (0.50–0.85)	Serum creatinine	1.928 (1.064–3.494) dL/mg	0.030
			INR	3.685 (1.093–12.426)	0.035
	Intraoperative	0.76 (0.55–0.87)	Area SBP < 100 mmHg	1.000 (0.999–1.000) (mmHg·min)^−1^	0.150
			Fresh frozen plasma	0.932 (0.843–1.031) kg/mL	0.170
			MAD SVI	1.394 (0.971–2.002) m^2^/mL	0.072
			RBC	1.186 (1.036–1.358) kg/mL	0.014
			Area CVP > 5 mmHg	1.000 (1.000–1.001) (mmHg·min)^−1^	0.100
	Combined	0.82 (0.66–0.94)	Area SpO_2_ < 90%	0.983 (0.941–1.026) (%·min)^−1^	0.423
			Serum direct bilirubin	2.834 (1.274–6.302) dL/mg	0.011
			MAD CVP	0.320 (0.116–0.879) mmHg^−1^	0.027
			Area CVP > 5 mmHg	1.001 (1.000–1.001) (mmHg·min)^−1^	0.005
			Serum total bilirubin	0.574 (0.323–1.019) dL/mg	0.058
	Blood product volumes and duration	0.72 (0.45–0.87)	Whole blood	1.117 (1.019–1.225) kg/mL	0.018
			Fresh frozen plasma	0.966 (0.896–1.042) kg/mL	0.374

AUC: area under the receiver operating characteristic curve; CI: confidence interval. For abbreviations in feature names, see Table 2.

Lastly, classifiers using only blood product volumes and surgery duration achieved a maximum AUC of 0.75 (95% CI: 0.56–0.93) (Table 3). This best classifier surpassed the best preoperative-features-only classifier (P = 0.04) and rivaled the best intraoperative-features-only (P = 0.26) and combined-features (P = 0.39) classifiers.

### Postoperative ARF

As with mortality prediction, intraoperative-features-only (maximum AUC = 0.76, 95% CI: 0.55–0.87) and combined-features classifiers (maximum AUC = 0.82, 95% CI: 0.66–0.94, P = 0.51) achieved the highest performances (Table 3). These did not, however, significantly outperform the best preoperative-features-only classifiers (maximum AUC = 0.72, 95% CI: 0.50–0.85, P = 0.68 and 0.32, respectively), two of which achieved AUC greater than 0.7.

In high-performing intraoperative-features-only classifiers, two features stand out with the greatest inclusion: whole blood volume administered and time-integrated area of the CVP signal above 5 mmHg (Fig. 2c). Similarly, but not as drastically, in high-performing classifiers with pre- and intraoperative features, serum direct bilirubin and time-integrated area of CVP above 5 mmHg stand out with most frequent inclusion (Fig. 2d).

Finally, ARF prediction with only blood product volumes and duration achieved a maximum AUC of 0.72 (95% CI: 0.45–0.87) (Table 3). This did not significantly differ from the best AUC of any other feature set (P = 0.98, P = 0.53, and P = 0.32 for comparison to preoperative-only, intraoperative-only, and pre- and intraoperative feature sets, respectively).

## Discussion

Risk stratification for adverse events following major surgeries remains a clinical challenge. Advances in accurate and minimally invasive hemodynamic monitoring provide the opportunity to use more detailed information to improve intraoperative management^23^. However, such data have traditionally been discarded without systematic analysis. In this study, we captured and archived these data by building dedicated hardware and software infrastructure at our center and training clinicians to use this system. We present evidence that the collected data contain important information that allows for improved prediction of postoperative events and therefore may guide prioritization of care resources following OLT, as well as, potentially, other high-risk surgeries.

Using real-time hemodynamic data to aid risk stratification is especially important given the limited value of preoperative data in predicting OLT outcomes in general, and mortality in particular. Systematic reviews of predictions based on the MELD score have shown an AUC for postoperative mortality that is consistently below 0.7^4,6^. Other preoperative scores, such as the Survival Outcomes Following Liver Transplantation score, also have comparatively low predictive abilities, with AUCs below 0.7^24,25^. In our study, we found a maximum AUC of 0.53 in predicting 180-day mortality with preoperative factors alone. By using features from hemodynamic variables and volumes of administered blood products, we achieved significantly better performance (AUC = 0.82, P = 0.001).

For postoperative ARF, elevated preoperative creatinine is an established risk factor^3^. However, even in combination with other preoperative measures, we achieved a classification ability that was only marginally above an AUC of 0.7. By adding intraoperative features, we achieved an improvement (though not statistically significant at P = 0.32) to a maximum AUC of 0.82.

Taken together, our results show that intraoperative variables contain useful information that aids in the prediction of OLT outcomes and significantly improves upon predictive performance when compared to using only preoperative variables. Hemodynamics have been used with data-driven approaches in other clinical settings to predict outcomes like mortality^26^. In various surgical contexts, prior evidence suggests that perioperative hemodynamic monitoring usage, particularly in high-risk cases, is associated with improvement of ARF, mortality, and infections^27–31^. Our work builds on these methods to perform risk stratification with intraoperative hemodynamic data in OLT and to potentially optimize perioperative care by targeting patients at greatest risk immediately after surgery.

Our findings also have implications for patient management. Previous studies of the effects of intraoperative hemodynamic management on OLT outcomes have largely derived results focused on ABP and CVP^7–15^. Even after starting with a sizable feature pool extracted from a large and diverse set of hemodynamic variables, we still found that CVP-related features in particular were significant predictors of outcome. For both mortality and ARF, the time-integrated area of CVP above 5 mmHg was the single most frequently included hemodynamic feature in the best-performing classifiers, with ORs suggesting that exposure to greater CVP is associated with occurrence of both outcomes (Fig. 2). While the magnitude of the OR may seem small (Table 3), it is important to recognize that for this continuous variable, the units indicate that the relative odds of, for example, 180-day mortality, increase by a factor of 1.001 for every 1 mmHg and every 1 minute that CVP is above 5 mmHg. For a long surgery such as OLT, the cumulative effect size can be quite large.

The harms and benefits of maintaining a low CVP are not agreed upon^18,19^, and our results seem to contradict one study that examined the 30-day postoperative mortality rates between a center that attempted to keep CVP below 5 mmHg in OLT patients as a matter of practice and another center that did not modify CVP^13^. However, it is unclear if CVP was actually lower in the nominal “low CVP” population. Furthermore, other studies have shown benefits for maintaining CVP below the 5 mmHg threshold or below some patient-specific baseline^11,12,32^. In contrast, our results show that a greater MAD of CVP is associated with reduced mortality and renal failure (Fig. 2). To the best of our knowledge, variability of CVP in OLT has not been studied before. It is therefore difficult to interpret and explain this finding. One of the studies describing the benefits of lower CVP described a CVP correction occurring during caval unclamping that may be associated with positive outcome^32^. Thus, in general, different CVP levels may be important in different phases of the surgery, and this may manifest as increased MAD of CVP.

In addition, we found other hemodynamic indices that could be useful predictors of outcome. Time-integrated area of SVI below 40 mL/m^2^ was the most frequently included non-CVP-related hemodynamic feature in high-performing classifiers for both mortality and ARF (Fig. 2). ORs indicate that exposure to reduced stroke volume is significantly associated with greater risk of both outcomes. Similarly, increased time-integrated exposure to HR > 100 bpm is also associated with greater risk of ARF. Other hemodynamic features with significant associations in high-performing classifiers were time-integrated areas of SVV and PPV above 10%. ORs of features derived from SVV and PPV (measures of fluid responsiveness^17^) show that OLT patients in whom SVV and PPV trend to levels that indicate fluid responsiveness and low-volume status experience greater occurrence of both 180-day mortality and ARF. These effects suggest that the patients who do best are those whose intraoperative course avoided intravascular volume depletion while minimizing exposure to CVP above 5 mmHg. Overall, these results demonstrate that a simple machine learning analysis of archived bedside monitoring data can help identify intraoperative monitoring variables that improve our ability to predict postoperative outcomes.

Our results also showed that administered blood product volumes were highly significant predictors of both outcomes, even on their own. The best 180-day mortality classifiers using only these features reached AUCs that significantly outperformed the best classifiers that used only preoperative features (AUC = 0.75 *vs*. AUC = 0.53, P = 0.035). Although these classifiers did not reach the performance of classifiers using intraoperative hemodynamic features in either mortality (AUC = 0.75 *vs*. AUC = 0.82) or ARF (AUC = 0.72 *vs*. AUC = 0.82), differences were not statistically significant (P = 0.26 and P = 0.32, respectively). Where infrastructure for collecting and storing intraoperative waveform signals for analysis is not available, readily available intraoperative clinical metrics appear to be reasonable surrogates, and their performance further emphasizes the benefit of using intraoperative information compared to using only preoperative information. From a data mining standpoint, though, using these surrogate metrics alone has important shortcomings when compared to using hemodynamic data. An OLT patient may receive a relatively greater amount of blood products for a variety of pre- and intraoperative reasons and risk factors, all of which generally indicate greater acuity^33,34,35^. We therefore used this small set of variables as a natural, if somewhat loose, summary metric of the overall difficulty of an OLT case. However, the multifactorial nature of the indication for intraoperative transfusions and the fact that transfusions are purely an intervention (rather than direct measurements of the patient’s physiology) make it challenging, if not impossible, to uncover specific or mechanistic processes behind adverse outcomes. In contrast, because hemodynamic variables are in principle modifiable through intraoperative management, it is possible to formulate specific hypotheses for how to actively intervene to improve – and not just predict – outcomes and even to motivate trials to test these hypotheses.

An important part of this work focused on addressing the problems of multicollinearity^21^ and low sample size relative to the number of features^22^ in logistic regression (see online Supplementary Material 2). In the contexts of OLT and other major surgeries, such considerations are critical for ensuring reliability. The process of archiving data is difficult and resource- and labor-intensive, and major surgeries may be performed infrequently at any given center. Consequently, the volume of data may not be “big” and standard “big-data” approaches thus not directly applicable. Indeed, to the best of our knowledge, our dataset of hemodynamic signals in OLT is the largest available world-wide. In this context, complex surgical procedures may stand to benefit the most from mining of intraoperative (and other) data. As similar analyses become more common, complex, and powerful, caution is necessary to account for data quantity and quality.

*Limitations:* This study has several limitations. Primarily, it is limited by its single-center, retrospective nature. Learning-based prediction can only be reasonably applied to a population similar to the training population, though it is important to note that the incidence of 180-day mortality here was similar to other Italian and European centers^36^. We recognize that exclusion of a substantial number of patient records has the potential to introduce sampling bias. Given that most excluded records were missing data from multiple input signals and that there was otherwise no clear pattern in the missing variables, we believe that inadvertent errors in operation of the data collection system were mostly responsible for the loss of data. We do not believe that these errors were systematic and therefore biased our results in a particular direction.

We attempted to optimize a trade-off between the included features and the potential for model-fitting errors due to feature multicollinearity. Our results could be affected by the choice of a different maximum acceptable condition number (see Methods – Subset selection), which determined the trade-off. We show in Fig. S2 (Supplementary Material 2) how this choice affects the results of subset selection. Substantially reducing the condition number could eliminate some important features (*e*.*g*., time-integrated area of CVP > 5 mmHg), while raising it would allow us to include other features that may improve apparent predictive performance, though potentially at the expense of model robustness. For example, blood transfusion volume was the single most included feature in predicting both mortality and ARF when using only intraoperative features, but was not included in the subset of combined pre- and intraoperative features. As more data become available, features left out by subset selection should be further investigated as they may further improve predictive performance without the penalty of reduced model robustness. Similarly, greater availability of data will enable use of more complex models, which can further leverage the richness of the hemodynamic data we have aggregated.

## Conclusion

Major surgeries like OLT can have high incidences of adverse postoperative events as well as significant inter-center variability in intraoperative care. Here, we took advantage of the rich set of monitoring data available during these types of surgeries to collect, archive, and mine a host of dynamic and continuous (often invasively acquired) intraoperative hemodynamic signals. Our work points to the potential that mining this information holds in improving outcome prediction. Through prudent feature selection and application of machine learning techniques, we demonstrate that inclusion of perioperative information can significantly improve risk stratification over use of preoperative data only, and we identify variables that may warrant further attention in studies on optimizing intraoperative management. While the predictive ability we have demonstrated here is encouraging, future validation on larger data sets, preferably from multiple clinical centers, is required to demonstrate predictive power and robustness at a level that aids in the clinical decision making process.

## Methods

This retrospective analysis was conducted on records from patients undergoing OLT at the University of Rome “Tor Vergata” between November, 2010 and March, 2016. Data collection was approved by the university’s Institutional Review Board, and all methods were performed in accordance with the Declaration of Helsinki.

### Data collection

Preoperative laboratory and clinical information was extracted from medical records (Table 1). Postoperative ARF was diagnosed according to the RIFLE criteria^37^. Postoperative mortality was established by the surgical team in routine postoperative follow-up. Intraoperative monitoring involved the S/5 Avance (GE Healthcare; Little Chalfont, UK) and PiCCO2 (Pulsion Medical Systems; Feldkirchen, Germany) hemodynamic monitors. Extraction and archiving of monitoring data were performed with custom software built in-house^38^. Full details of intraoperative instrumentation and data collection are in previous work^38–40^ and online Supplementary Material 1. Analysis proceeded as follows (Fig. 1a).

### Feature extraction

We extracted fifteen preoperative and 41 intraoperative features (Table 2). Preoperative features included laboratory values commonly recorded in OLT patients immediately before surgery as well as demographics and certain comorbidities. Intraoperative features included volumes of four different blood products administered (normalized to body mass), total duration of the surgery, and hemodynamic features extracted from the signals and indices recorded and computed by the bedside monitors. Hemodynamic features included each variable’s median and, for variables recorded as continuous time-series (in contrast with those computed only intermittently), the median absolute deviation (MAD) of the time-series and integrated area of the portions either below or above the nominally “normal” threshold provided by the device manufacturer (Fig. 1b). This latter “exposure area” feature provides a measure of total integrated exposure to these putatively dangerous conditions. Further details about normal thresholds are provided in online Supplementary Material 1, Table S1.

### Inclusion criteria

In total, 101 records were reviewed, including 14,291 overall hours of intraoperative waveform data. Two re-transplant cases were excluded in favor of including those patients’ original cases only. Individual records were also excluded if any preoperative value was absent, if any hemodynamic signal contained fewer than 180 minutes of data, or if any administered blood product volume was missing – *i*.*e*., presence of all 56 features and relevant outcome information was required for inclusion of a patient record. In total, 62 records were available for prediction of both 180-day mortality and ARF. Among excluded records, 10 were missing at least one preoperative variable, 23 were missing data from at least one hemodynamic signal (13 of which were missing more 2 or more), and 14 were missing blood product volume administration measurements (13 of which were also missing 2 or more). Thirty-eight records in total were excluded for missing input data, with 24 missing two or more preoperative variables, hemodynamic signals, or blood product volumes. The remainder of patients were excluded due to incomplete follow-up for outcome information. The most commonly missing input variable was CVP (missing in 16 patients), followed by platelet, fresh frozen plasma, and whole blood transfusion volumes (13 patients), and SVV (missing in 11 patients).

### Prediction methods

As described earlier, four binary prediction tasks were performed for each outcome, where each task differed by the set of features used (all features, preoperative features only, intraoperative features only, and lastly, non-hemodynamic intraoperative features only). Within an individual outcome, the same set of patient records was used in all tasks.

### Subset selection

To reduce the overall number of features and feature combinations searched and the potential multicollinearity among them, we used a subset selection procedure in each task that was blinded to the true class labels. We first constructed a two-dimensional feature matrix in which the rows represent the different patients in our cohort and the columns represent the values of the different features. The subset selection procedure then used the QR decomposition of the feature matrix with column pivoting^41^. Briefly, this numerical method decomposes the feature matrix into a product of an orthogonal matrix (the “Q” matrix) and an upper-triangular matrix (the “R” matrix). The column-pivoting steps determine a permutation matrix, P, which reorders the columns (*i*.*e*., the features) of the feature matrix such that the magnitudes of the diagonal elements of the R matrix are  non-increasing. The first k columns of the reordered feature matrix then represent the single k-sized subset of columns that is maximally linearly independent. This reorganization therefore reflects a trade-off between the amount of information captured and the risk of including redundant features (multicollinearity).

To choose the size of a subset, we selected the largest number of features that formed a matrix with condition number no greater than 15. The condition number of a matrix in generalized linear regression models describes the extent to which errors in the input data cause errors in the output data, and is strongly affected by multicollinearity. A maximum condition number of 15 was therefore chosen *a priori* to place a limit on the extent of multicollinearity among the selected variables^21^. While there is no universally agreed upon maximum acceptable condition number, our choice of 15 represents a compromise within a commonly recommended range of 10–20^21,42^. In this way, we attempted to maximize the amount of information retained after excluding features while minimizing problems associated with multicollinearity.

Subset selection was performed after standardizing the feature matrix by subtracting out and dividing by the mean value of the feature across patients. All patient records possessing relevant data were used, irrespective of the presence of outcome information, allowing the same feature subsets to be used for both outcomes.

### Binary outcome prediction

After choosing this subset of features, we used logistic regression with all possible combinations of up to five of these features to find the best performing sets of features. This limit was imposed to limit the ratio of features to mortality or ARF cases, as a larger ratio can reduce the reliability of logistic regression coefficient estimates^22^.

Using a leave-one-out cross-validation (LOOCV) procedure, each record was classified into either the outcome or control group by a logistic regression classifier trained on all other records. This was repeated with every unique combination of five or fewer features. LOOCV is a reasonable alternative to testing performance on a hold-out test set of records not used for training when only a small number of records is available^43^. Multivariate odds ratios (ORs) were computed from regression coefficients.

Performance was evaluated by the area under the receiver operating characteristic curve (AUC), constructed by varying a threshold on the predicted probability of suffering the selected outcome, as computed by logistic regression. Confidence intervals (CIs) at 95% were computed by the bootstrap bias correction and acceleration method with 1,000 replicates^44^ using the MATLAB Statistics Toolbox (The Mathworks, Inc; Natick, MA, USA). Within outcomes, the best AUCs achieved in each task were statistically compared pairwise following DeLong *et al*.^45^, considering P < 0.05 statistically significant.

### Ethics approval

This study was approved by the Institutional Review Board (IRB) of the University of Rome “Tor Vergata” (study number 24516).

### Informed consent

Need for consent was waived by the IRB of the University of Rome “Tor Vergata” (study number 24516).

### Availability of data and materials

The datasets used and analyzed during the current study are available from the corresponding author on reasonable request.

## Electronic supplementary material

Supplementary Information
