# Supplementary material for: Prediction of postoperative outcomes using intraoperative hemodynamic monitoring data
Source: Sci Rep. 2017 Nov 27;7:16376. doi: 10.1038/s41598-017-16233-4 (PMC5703992; doi:10.1038/s41598-017-16233-4)
Supplement: Supplementary file 1 — Supplementary Information [file 41598_2017_16233_MOESM1_ESM.pdf]

SUPPLEMENTARY MATERIALS

PREDICTION OF POSTOPERATIVE OUTCOMES USING INTRAOPERATIVE HEMODYNAMIC MONITORING DATA

Varesh Prasad, BSE, Maria Guerrisi, PhD, Mario Dauri, MD, Filadelfo Coniglione, MD, Giuseppe Tisone, MD, Elisa De Carolis, MD, Annagrazia Cillis, MD, Antonio Canichella, Nicola Toschi, PhD, Thomas Heldt, PhD.

Supplementary Material 1: Intraoperative Data Collection and Intraoperative Features

Intraoperative Data Collection: Instrumentation of patients during surgery followed standard clinically indicated protocols and was arranged for monitoring of hemodynamic and respiratory signals, including arterial blood pressure (ABP), central venous pressure (CVP), electrocardiogram (ECG), air flow (AF), and air pressure (AP). The ECG, AF and AP signals were measured with standard transducers and monitored by an S/5 Avance bedside monitor (GE Healthcare; Little Chalfont, United Kingdom) that provided digital output of the ECG at 300 Hz and of AF and AP at 25 Hz. ABP was measured with an invasive line located in the right brachial artery, while CVP was measured with a central venous line inserted through the right jugular vein and advanced to the superior vena cava. ABP and CVP signals were monitored by a PiCCO2 hemodynamic monitor (Pulsion Medical Systems; Feldkirchen, Germany), which provided these signals at 100 Hz. The PiCCO2 monitor separated these signals into systolic and diastolic trend components at 0.1 Hz and also computed several other hemodynamic indices, some at 2.5 Hz and others only intermittently, as requested by care providers (Table 2). The ABP waveform was acquired redundantly on both the PiCCO2 and S/5 devices and used in post-processing to time-align the data streams from both monitors.

To request and unpack data from both monitors, custom acquisition software was built and integrated into a single software application, called Global Collect (GC). GC was developed in-house[1] in the LabVIEW environment (National Instruments Corp.; Austin, TX, USA) and interfaces with different patient monitoring devices though an RS232-USB2.0 hub and a National Instruments NI USB-6008 board, allowing real-time acquisition, visualization, processing, and archiving of high-resolution waveform and trend data (termed "physiological data" on GE monitors).

A list of all the signals archived from the monitors and analyzed here is provided in Table S1. Time-series from continuous variables were 5-point median filtered to remove outliers before feature extraction. Wherever possible, we used variables normalized to body surface area or body weight. We did not make use of normal ranges for variables computed only intermittently, and so the ranges for these variables are not listed here.

For several of the indices, no threshold was provided: For systolic ABP, SpO_2, and HR, we used the generally accepted thresholds of 100 mmHg, 90%, and 100 bpm, respectively. For CVP, we used 5 mmHg as the upper threshold believed to be useful in preventing substantial blood loss[2–4]. Lastly, for dPmx, we used the 33rd percentile of all observed data points from all patients' available data as an empirical threshold for poor cardiac contractility.

Table S1: List of analyzed hemodynamic signals and associated information

Signal or index (abbreviation) [units]	Recording device	Temporal resolution (samples/second)	Normal range used
**Systolic arterial blood pressure (SBP) [mmHg]**	Avance S/5	0.1	< 100 mmHg
**Central venous pressure (CVP) [mmHg]**	Avance S/5	0.1	< 5 mmHg
**Heart rate (HR) [bpm]**	Avance S/5	0.1	< 100 bpm
**Peripheral oxygen saturation (SpO_2) [%]**	Avance S/5	0.1	> 90%
**Cardiac function index (CFI) [min^{-1}]**	PiCCO2	Intermittent	-
**Max left ventricular contractility (dPmx) [mmHg/s]**	PiCCO2	2.5	< 642 mmHg/s
**Extravascular lung water index (ELWI) [mL/kg]**	PiCCO2	Intermittent	-
**Global end-diastolic volume index (GEDI) [mL/m^2]**	PiCCO2	Intermittent	-
**Global ejection fraction (GEF) [%]**	PiCCO2	Intermittent	-
**Intrathoracic blood volume index (ITBI) [$mL/min/m^2$]**	PiCCO2	Intermittent	-
**Pulse-contour cardiac index (PCCI) [$L/min/m^2$]**	PiCCO2	2.5	< 3 $L/min/m^2$
**Pulse pressure variation (PPV) [%]**	PiCCO2	2.5	< 10%
**Pulmonary vascular permeability index (PVPI)**	PiCCO2	Intermittent	-
**Stroke volume index (SVI) [mL/m^2]**	PiCCO2	2.5	< 40 mL/m^2
**Systemic vascular resistance index (SVRI) [$dyn \cdot s \cdot cm^{-5} \cdot m^2$]**	PiCCO2	2.5	< 1700 $dyn \cdot s \cdot cm^{-5} \cdot m^2$
**Stroke volume variation (SVV) [%]**	PiCCO2	2.5	< 10%

References

1. Toschi, N. *et al.* Intraoperative haemodynamic monitoring: A pilot study on integrated data collection, processing and modelling for extracting vital signs and beyond. in *2010 3rd International Symposium on Applied Sciences in Biomedical and Communication Technologies (ISABEL 2010)* 1–5 (IEEE, 2010). doi:10.1109/ISABEL.2010.5702766
2. Feng, Z.-Y., Xu, X., Zhu, S.-M., Bein, B. & Zheng, S.-S. Effects of low central venous pressure during preanhepatic phase on blood loss and liver and renal function in liver transplantation. *World J. Surg.* **34,** 1864–1873 (2010).
3. Schroeder, R. A. *et al.* Intraoperative fluid management during orthotopic liver transplantation. *J. Cardiothorac. Vasc. Anesth.* **18,** 438–441 (2004).
4. Wang, B., He, H., Cheng, B., Wei, K. & Min, S. Effect of low central venous pressure on postoperative pulmonary complications in patients undergoing liver transplantation. *Surg. Today* **43,** 777–781 (2013).

Supplementary Material 2: Feature subset selection results

Figure S2 shows how the condition number increases as more features are included. In experiments with only preoperative features, eleven features formed the largest matrix with condition number less than or equal to 15 (Fig. S2a). In experiments with only intraoperative features, the limit was met at twenty-two features (Fig. S2b), and in experiments with both pre- and intraoperative features, the limit was met at twenty-seven features (Fig. S2c). Within the combined set, 10 of the 27 features were preoperative features and the remaining 17 were intraoperative, including three blood product volumes. Limiting the number of features included in any one classifier to five resulted in totals of 1,023 combinations of only preoperative features, 35,442 combinations of only intraoperative features, and 101,583 combinations of pre- and intraoperative features.

Our overall results did not reveal clear evidence of errors due to multicollinearity or low relative event number: Individual features showed a significant association with outcome with consistency in the same direction in all classifiers (Fig. 2). Furthermore, significant ORs had consistent values when used in different experiments for the same outcome. For example, in predicting mortality, MAD dPmx had an OR of 0.987 s/mmHg (95% CI: 0.968-1.007 s/mmHg) in a classifier that used only intraoperative features and a very similar OR of 0.989 s/mmHg (95% CI: 0.968-1.010 s/mmHg) in a classifier that used both pre- and intraoperative features.

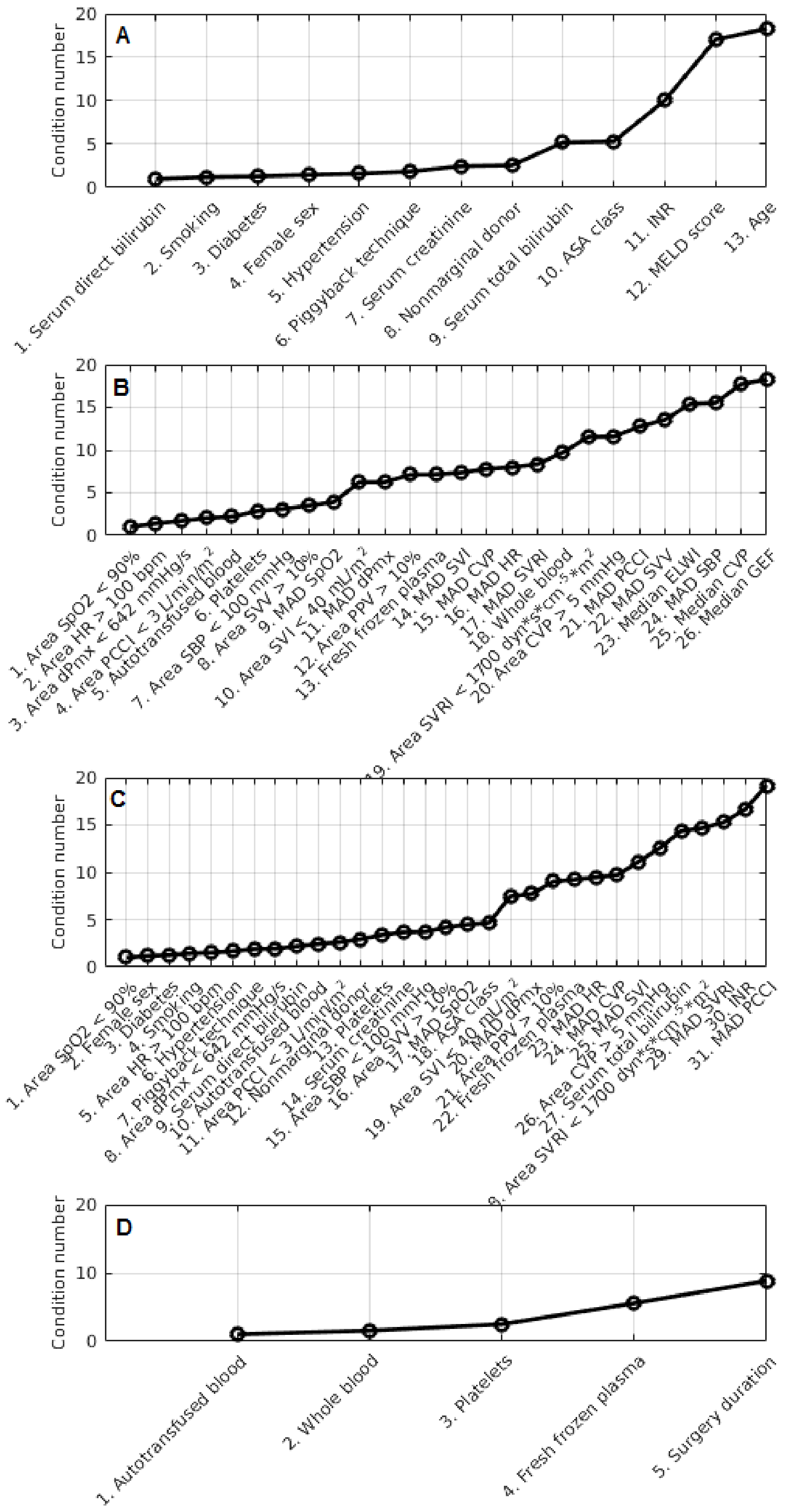

Figure S2. Condition numbers of feature matrices as features are added back after QR decomposition with column pivoting. **A** (top): Preoperative features only. **B:** Intraoperative features only. First 26 features, up to a condition number of 20, are shown for brevity. **C:** Pre- and intraoperative features combined. First 31 features, up to condition number of 20, are shown for brevity. **D** (bottom): Blood products only. All five features are shown. In each case, the largest subset of features with a condition number less than or equal to 15 was selected for further analysis.
